# A Phage Tail-Derived Element with Wide Distribution among Both Prokaryotic Domains: A Comparative Genomic and Phylogenetic Study

**DOI:** 10.1093/gbe/evu136

**Published:** 2014-07-11

**Authors:** Panagiotis F. Sarris, Emmanuel D. Ladoukakis, Nickolas J. Panopoulos, Effie V. Scoulica

**Affiliations:** ^1^Laboratory of Clinical Bacteriology and Molecular Microbiology, Faculty of Medicine, University of Crete, Heraklion, Greece; ^2^Department of Biology, University of Crete, Heraklion, Greece; ^3^Department of Plant Pathology and Microbiology, Center for Plant Cell Biology and Institute for Integrative Genome Biology, University of California, Riverside; ^4^Present address: The Sainsbury Laboratory, John Innes Centre, Norwich Research Park, Norwich, United Kingdom.

**Keywords:** bacterial protein translocation, PLTS, T6SS, PVC, Afp, phage tail-like element

## Abstract

Prophage sequences became an integral part of bacterial genomes as a consequence of coevolution, encoding fitness or virulence factors. Such roles have been attributed to phage-derived elements identified in several Gram-negative species: The type VI secretion system (T6SS), the R- and F-type pyocins, and the newly discovered *Serratia entomophila* antifeeding prophage (Afp), and the *Photorhabdus luminescens* virulence cassette (PVC). In this study, we provide evidence that remarkably conserved gene clusters, homologous to Afp/PVC, are not restricted to Gram-negative bacteria but are widespread throughout all prokaryotes including the Archaea. Even though they are phylogenetically closer to pyocins, they share key characteristics in common with the T6SS, such as the use of a chaperon-type AAA+ ATPase and the lack of a host cell lysis mechanism. We thus suggest that Afp/PVC-like elements could be classified as phage-like-protein-translocation structures (PLTSs) rather than as pyocins. The reconstruction of phylogeny and the conserved gene content suggest that the diversification of prophage sequences to PLTS occurred in bacteria early in evolution and only once, but PLTS clusters have been horizontally transferred to some of the bacterial lineages and to the Archaea. The adaptation of this element in such a wide host range is suggestive of its versatile use in prokaryotes.

## Introduction

Modules or genes from tailed phages have evolved to become fundamental components of bacterial machineries such as secretion systems or pyocins (Veesler and Cambillau 2011). From these bacterial systems, the ones that are reminiscent of bacteriophage contractile tails in terms of morphology, size, and even function are the type VI secretion system (T6SS) (Leiman et al. 2009; Bönemann et al. 2010), the R- and F-type pyocins (Nakayama et al. 2000; Michel-Briand and Baysse 2002), and the recently identified needle-like particles, the antifeeding prophage (Afp) from *Serratia entomophila* (Hurst et al. 2004) and the virulence cluster from *Photorhabdus luminescens* (PVC) (Yang et al. 2006). These elements have been detected so far only in Gram-negative bacteria and are functionally distinct: R- and F-type pyocins have the role of membrane attack against closely related bacteria (Iijima 1978; Uratani and Hoshino 1984), whereas T6SS has a dual role: It attacks bacterial cells (Hood et al. 2010) but is also able to induce morphological changes in the cytoskeleton of eukaryotic cells (Ma and Mekalanos 2010; MacIntyre et al. 2010). Afp/PVC were initially grouped with R-type pyocins due to their closer genetic similarity; however, functional studies revealed that they confer toxicity toward insect hemocytes by inducing actin condensation (Yang et al. 2006). Two other independent studies demonstrated the presence of fibril-like structures in two unrelated bacterial species: 1) *Cardinium hertigii*, a member of the Bacteroidetes and symbiont of the parasitic wasp *Encarsia pergandiella* (Penz et al. 2012), and 2) *Saprospira* SS98-5, a Gram-negative bacterium that exhibits algicidal activity through direct attack and, moreover, is able to prey on *Chaetoceros ceratosporum*, a eukaryotic bacillariophyte (Furusawa et al. 2005). Interestingly, the gene clusters encoding for the fibril-like structures in both species appear to be related to Afp/PVC.

The architecture of these tail-like bacterial machineries maintains the basic organization of the contractile phage tail consisting of the main tube, the outer sheath, and the baseplate all of which are encoded on discrete chromosomal loci (Nakayama et al. 2000; Sen et al. 2010; Heymann et al. 2013).

The T6SS, R-type, and F-type pyocins share an ancestral origin with T4, P2, and λ phages, respectively, and they are restricted to Gram-negative bacteria, the natural hosts of these phages. Gram-positive bacteria are known to host-tailed phages of the *Myoviridae* family, characterized by their contractile tails (Ackermann 2009); however, no phage-like particles have been identified until now, the only possible exception to this being the electron microscopy observation in some *Streptomyces* species, of hexameric membrane structures linked intracellularly with contractile tail-like particles (Ogata et al. 1982). These structures, termed pocks, are plasmid encoded and were attributed the role of plaque formation and bacterial lysis during cell growth on solid material. Archaeal viruses have been identified to infect members of the *Euryarchaeota* and *Crenarchaeota* phyla, but they do not share sequence similarity with bacterial phages (Krupovic et al. 2012) and there are no reports known to date on their phylogenetic relation to prokaryotic machineries.

In this study, we show that gene clusters homologous to Afp/PVC (hereafter called PLTS, phage-like-protein-translocation structure) are conserved in sequence and gene order within the genomic context of phylogenetically distinct Gram-negative, Gram-positive bacterial and in archaeal genomes. We present phylogenetic evidence revealing that the phage tail-like components of the PLTS gene clusters share a common ancestor with the corresponding structural components of T6SS and R-type pyocins. Comparative analysis of PLTS gene content with components of T6SS and pyocins, for which functional data are available, revealed molecular characteristics that are informative for the role of this largely unknown element in prokaryotes.

## Results and Discussion

Comparative analysis of whole-microbial genome sequences hosted by National Center for Biotechnology Information (NCBI) enabled the identification of a conserved gene cluster, homologous to Afp/PVC. The 13–16 genes, identified in this cluster were found within hundreds of prokaryotic genomes from bacterial and Archaeal domains (table 1), originating from a variety of environmental niches ranging from free-living soil bacteria, to plant and animal symbionts.

**Table 1:** Taxonomy Report of the Organisms that Harbor PLTS Loci

Domain	Phylum	Class/order/family	Number of Organisms	Position of the PLTS Cluster in the Genome of the Representative Strains Used in This Study
Bacteria			431	
	Actinobacteria		137	
		Corynebacterineae	6	*Mycobacterium* sp. JLS: Mjls_0922-0939
		Frankineae	6	*Nakamurella multipartite*: Namu_1892-1910
				*Frankia* sp. EAN1pec: Franean1_3810-3826
		Propionibacterineae	4	*Microlunatus phosphovorus*: MLP_03590-03730
		Micromonosporaceae	12	
		Streptomycineae	74	*Streptomyces scabiei*: Scab_27561-27711
				*Streptomyces coelicolor*: Sco4243-4260
		Catenulisporineae	2	*Catenulispora acidiphila*: Caci_4397-4383
		Pseudonocardineae	18	*Actinosynnema mirum*: Amir_5380-5394
				*Amycolatopsis mediterranei* U32: Amed_4831-4846
		Micrococcineae	11	*Cellulomonas flavigena*: Cfla_2257-2270
		Acidimicrobium	2	
		Ilumatobacter	2	
	Firmicutes		22	
		Paenibacillaceae	8	*Brevibacillus brevis*: Bbr47_4220-42-70
		Clostridiales	8	*Clostridium symbiosum*: HMPREF9474_03843-3856
		Treponema	4	
		Spirochaeta	2	
	Roseiflexus		3	*Roseiflexus* sp. RS-1: RoseRS_1650-1667
				*Roseiflexus castenholzii*: Rcas_2813-2831
	Deinococcus		9	*Deinococcus maricopensis*: Deima_3025-3037
	Proteobacteria		85	
		Alphaproteobacteria	16	*Erythrobacter litoralis*: Eli_09275-09370
		Deltaproteobacteria	23	
		Myxoccaceae		*Myxococcus xanthus*: Mxan_4492-4510
		Cystobacterineae		*Corallococcus coralloides:* Cocor_02848-02867
		Betaproteobacteria	11	
		Rhodocyclaceae		*Thauera* sp. MZ1T: Tmz1t_1864-1884
		Burkholderiaceae		*Burkholderia rhizoxinica*: Rbrh_00107-00122
		Gammaproteobacteria	35	
		Enterobacteriaceae		*Photorhabdus luminescens*: plu1692-1708
				*Serratia entomophyla*: Afp1-Afp14
				*Yersinia ruckeri*: yruck0001_1580-1730
		Shewanellaceae		*Shewanella denitrificans*: Sden_2996-3009
		Alteromonadaceae		*Saccharophagus degradans*: Sde_1036-1049
	Bacteroidetes–Chlorobi		85	
		Chlorobaculum	2	*Chlorobaculum parvum* NCIB 8327: Cpar_0887-0903
		Bacteroidetes	83	
		Sphingobacteriaceae		*Chitinophaga pinensis*: Cpin_3920-3935
		Bacteroidaceae		*Bacteroides fragilis* CL05T12C13: HMPREF-1080_04193-04207
		Flavobacteriaceae		*Leeuwenhoekiella blandensis*: MED217_08560-08650
	Cyanobacteria		52	
		Nostocales	14	*Nostoc punctiforme*: Npun_F1414-R1398
		Oscillatoriophycideae	34	*Cyanothece* sp. PCC 7822: Cyan7822_4448-4453, Cyan7822_1930-1931
				*Acaryochloris marina*: AM1_6406-6403, AM1_0270-0276
		Gloeobacteriales	2	*Gleobacter violaceus* PCC 7421: glr4099-4106, gll-425-0430, gll1410-1413
		Oscillatoriales		*Microcoleus* sp. PCC 7113: Mic7113_5982-5992
		Pleurocapsa	1	
		Chlorogloeopsis	1	
	Gemmatimonas		2	
	Planctomycete		1	
Archaea			35	
		Uncultured archaeon	1	
		Euryarchaeota	34	
		Halobacteriales		*Natrialba magadii*: Nmag_0754-0774
				*Halogeometricum borinquense*: Hbor_38920-38640
				*Natrinema* sp. J7-2: Nj7g_0009-0033
		Methanosarcinales		*Methanomethylovorans hollandica*: Metho_1612-1628

Note.—The organisms are sorted by BLAST in NCBI data library using the main structural gene products: Baseplate J, Sheath, VGRG, and GP25/W. PLTS-genes’ locus numbers are figured for the organisms used in the study as they are annotated in Kegg database (Kanehisa et al. 2014).

**Fig. 1.—:**
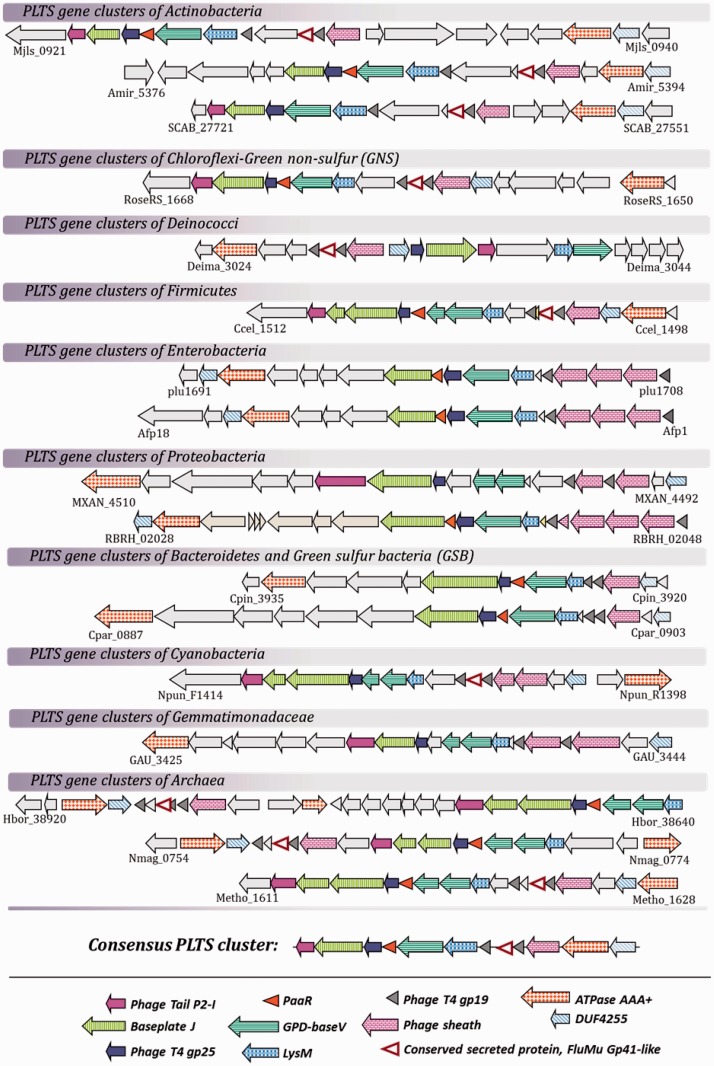
PLTS clusters found in representative genomes of Bacteria and Archaea and the deduced consensus cluster. The conserved genes that are present in all PLTS clusters studied in this report are shown marked in colors, with the exception of PAAR-motif and Mu-gp41-like encoding ORFs that are present only in a number of the examined bacterial genomes. The taxonomic report of the bacterial loci presented in this figure is included in table 1. Gene locus numbers have been included below the first and last ORF of each representative gene cluster.

On the basis of domain annotation and sequence similarity, we were able to define a core cluster of structural genes and their syntenic order, which displayed a remarkable level of conservation within bacterial and Archaea phyla (fig. 1). Within the main gene cluster, we observed a conserved subcluster comprising the genes of baseplate module assembly. This subcluster consists of orthologs of the T4 gene product 6 and 25 (gp6 and gp25) and P2 gpI baseplate-wedge components, preceded by the *vgrG* gene that harbors the domains of the baseplate-hub gp27/gp5, and by an open reading frame (ORF) with a C-terminal LysM domain (lysine motif), which is known to mediate peptidoglycan binding (Buist et al. 2008). In virtually all instances, the observed baseplate module harbors an ORF containing a proline–alanine–alanine–arginine (PAAR) repeat. PAAR-repeat proteins are essential for T6SS function because they form the sharp cone on the spike of T6SS, which pierces target cell membranes and are able to attach various toxic effectors (Shneider et al. 2013). It is noteworthy that PAAR-motif bearing proteins are a common characteristic of PLTS and T6SS but are absent from the pyocin gene clusters. The synteny of the baseplate structural components LysM, VgrG (gp27-gp5), PAAR, gp25, Baseplate J (gp6), and P2-I is remarkably conserved in the majority of the PLTS clusters, suggesting coregulation and defined order of attachment during baseplate assembly. The tube proteins are encoded downstream of the baseplate assembly genes and generally comprise two copies of the T4 gp19 homolog, along with a single copy of the gene which encodes the outer sheath protein. PLTS clusters always encompass a Walker A,B motif-containing chaperon-like AAA^+^ ATPase, which is usually associated with the assembly/disassembly of protein complexes. The position of these elements within the cluster, however, often varies relative to the baseplate subcluster. This type of ATPase is also associated with T6SS and acts in recycling the injection apparatus after a single use (Bönemann et al. 2009; Pietrosiuk et al. 2011). The ATPase gene itself frequently being located in a “head-to-tail” orientation with a gene coding for a protein characterized in the Pfam database as DUF4255. This gene presents homologies to the T4-gp3 gene, which codes for a sheath-stabilizing protein. Interestingly, it was recently found that the orthologous gene of the Afp element in *S. entomophila* is encoding for length determination and sheath stabilization protein (Rybakova et al. 2013). Table 2 lists the main components of the PLTS cluster and their homologs in R-type pyocins, T6SS, and T4 phage.

**Table 2:** The Minimal Set of Proteins Comprising the Consensus PLTS Cluster and the Orthologs of T6SS and R-Type Pyocins

Structural/Functional Components	Number of Genes or Domains in Plts	T4/P2	PLTS	T6SS	R-Pyocin
Baseplate hub (VgrG)	1	Gp27/gpD	GPD-baseV domains on VgrG protein or distinct polypeptides	GPD-baseV domains on VgrG protein	GPD
Spike (VgrG)	1	Gp5/gpV			baseV
Lysozyme (PG binding)	1	Lysozyme full activity	LysM-motif PG binding	ND	ND
Lysozyme (hydrolase activity)			—		
Tip of the spike	1	PAAR repeats	PAAR repeats	PAAR repeats	NF
Baseplate structural proteins	1–2	Gp6/gpJ	Baseplate J	ND	Baseplate J
	1	Gp25/gpW	Gp25/gpW	Gp25/gpW (TssE)	Gp25/gpW
	1–2	—/gpI	P2-I	ND	P2-I
Tail tube initiator		Gp48(54)/gpU	ND	ND	gpU
Tail tube	1–2	Gp19/gpFII	Gp19	Hcp	Tube
Sheath	1–3	Gp18/gpFI	Sheath	Sheath (TssB-TssC)	gpFI homolog
Tail fiber proteins		Gp12/gpH	ND	ND	Tail fiber protein
		Gp36 and gp37	ND	ND	Gp36 and gp37 homologs
Tail fiber assembly	1	Gp3	gp3-like	ND	Tail determination
Lysis “will out”		Holin	ND	ND	Holin
Helicase		Gp41	ND	ND	Gp41 homolog
AAA+ ATPase	1	—	ΑΑΑ+ ATPase	CLIP V	ND

Note.—The gene names were normalized with the homologous phage-gene names as they are annotated in ViralZone data library (viralzone.expasy.org). Because of high-sequence diversity and lack of functional data, nonidentified genes are marked as nondetermined (ND). NF, not found.

Although the modular organization of the PLTS gene cluster is conserved, we observed distinct characteristics within some prokaryotic families. We noticed a variable number of ORFs encoding the sheath, which in most microorganisms is encoded by one ORF, in Myxococcaceae, Cyanobacteria, and *Gemmatimonas* is encoded by two ORFs, and in Enterobacteriaceae is encoded by three phylogenetically distinct ORFs. Significant heterogeneity (i.e., sequence, gene size, and gene number) was specifically observed in the ORFs encoding for the Baseplate J protein. In Archaea, Cyanobacteria, and Firmicutes, this component is encoded by two distinct genes, whereas in Actinobacteria, Deinococci, and Chloroflexi, it appears to be encoded by a single, shorter ORF. Similarly, phage late control gene D protein (GPD) and base V domains usually encoded by a single *vgrG*-like *rhs*-element are encoded on separate syntenic ORFs in Archaea, Firmicutes*,* Cyanobacteria, *Gemmatimonas*, and Proteobacteria (with the exception of Enterobacteriaceae). Finally, the ORF coding for a PAAR-repeat containing protein appears to be absent from Deinococci, Proteobacteria, and some Cyanobacteria.

We notice, also, the following two features of particular interest: First, the chaperon-type AAA+ ATPase appears as a fundamental characteristic of both T6SS and PLTS as opposed to phages that harbor a DNA-dependent ATPase, whereas pyocins do not appear to contain a homolog to either of these genes. Second, the lack of a host cell lysis system in both PLTS and T6SS. Most phages accomplish host lysis using a muralytic enzyme and a holin, which permeates the membrane of the host cell at a programmed time (Young 2013). This latter step being crucial for phage release from the host appears to have been adopted by the pyocins, because they also contain an endolysin/holin lysis system but is absent in PLTS and T6SS. Although the LysM-motif bearing ORF is highly conserved in sequence and position within PLTS clusters, the absence of domains exhibiting hydrolase activity consequently suggests that this gene product is uniquely dedicated to peptidoglycan binding (Buist et al. 2008). We suggest that these two features functionally group together the PLTS and the T6SS and are consistent with a dynamic mechanism of effector translocation to target cells.

**Fig. 2.—:**
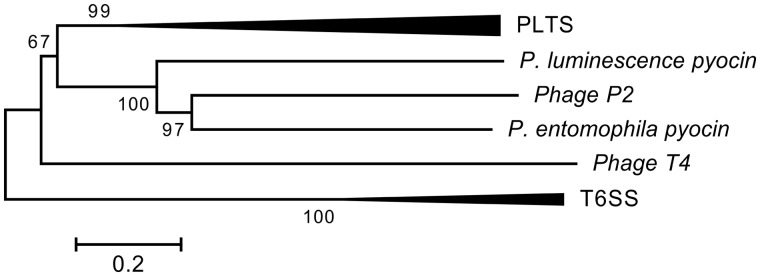
Phylogenetic relationships of PLTSs in relation with R-type pyocins, P2 and T4 phages, and T6SS. The evolutionary relationships between PLTSs, R-type pyocins, T4 and P2 phages, and T6SS have been constructed using concatenated protein sequences of three common main structural components: gp25, VgrG, and Sheath. The R-type pyocin orthologs are *Photorhabdus luminescens*: plu3425, plu3427, plu3426, and plu3432; and *Pseudomonas entomophila*: PSEEN4162, PSEEN4149, PSEEN4163, and PSEEN4155. The T6SS sequences used were *Photorhabdus asymbiotica*: PAU_00286, PAU_00273, PAU_00288, and PAU_00287; *Pectobacterium atrosepticum*: ECA3443, ECA3427, ECA3445, and ECA3444; *P. asymbiotica*: PAU_00286, PAU_00273, PAU_00288, and PAU_00287; and *P. entomophila*: PSEEN0525, PSEEN0540, PSEEN0523, and PSEEN0523. The corresponding phage protein sequences were retrieved from ViralZone data library (viralzone.expasy.org).

**Fig. 3.—:**
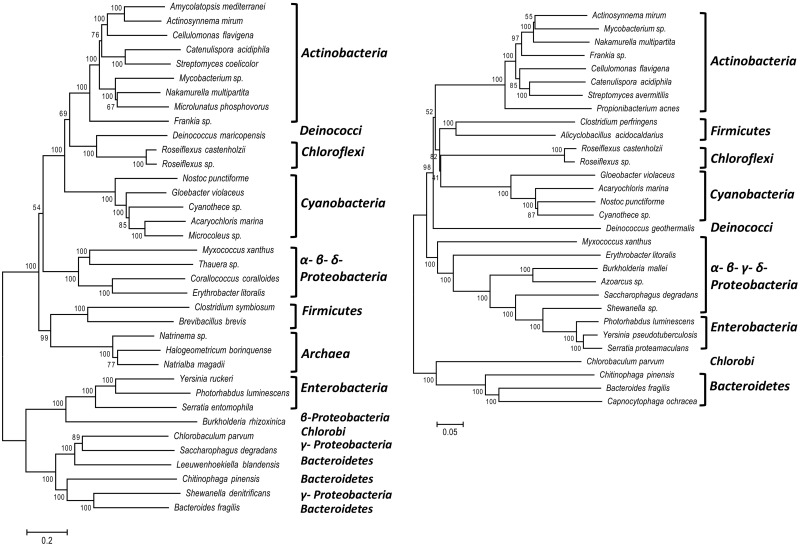
(*A*) Phylogenetic relationships of the PLTS clusters among prokaryotes based on five concatenated structural gene products. All the PLTS clusters used are listed in table 1. (*B*) Phylogenetic relationships among the bacteria which carry PLTS, based on 31 concatenated gene products.

Furthermore, we examined the evolutionary relations between PLTSs, R-type pyocins, T4 and P2 phages, and T6SS by constructing a phylogenetic tree using concatenated protein sequences of the three main structural components: gp25, VgrG, and Sheath (fig. 2). We observed that PLTSs and Afp/PVC form a monophyletic group, closer to pyocins than to T6SS. Having also reconstructed the phylogeny of the PLTS gene cluster across prokaryotes using concatenated protein sequences of five structural gene products: gp25, VgrG, Sheath, Baseplate J, and LysM (fig. 3*A*), we also observed that the major phylogenetic groups in the PLTS tree formed very distinct clusters (i.e., Actinobacteria, Chloroflexi, Cyanobacteria, Firmicutes, Enterobacteria, and Archaea). The relative order of these clusters agreed with the phylogeny of bacteria (fig. 3*B*) with the exception of Proteobacteria, Firmicutes, and Archaea as discussed below. This observation along with the fact that the conserved gene content of all PLTS examined originated from T4, P2, and λ phages consequently suggests that the diversification of PLTS in bacteria from prophage sequences is an ancient phenomenon which might have occurred only once. The alternative hypothesis that the diversification of PLTS in bacteria has occurred multiple times is less parsimonious and poorly supported by the data. However, it should be noted that there was no full agreement between the phylogeny of the main PLTS components and the phylogeny of the bacteria that carry the PLTS cluster, implying that the PLTS has been horizontally transferred among closely or distantly related prokaryotic lineages. First, the group of Archaea is found within the bacterial group in the PLTS tree, neighboring Firmicutes. In full prokaryotic phylogeny, however, the Archaea group always appears as an outgroup in the bacterial lineage (Pace 1997), implicating that Archaea have acquired their PLTS horizontally from Bacteria. Second, in the PLTS tree, the Firmicutes lineage is clustered with the Archaea, whereas in the full bacterial phylogeny, it is clustered with Chloroflexi and Cyanobacteria. This therefore suggests that Archaea and Firmicutes have both acquired their PLTS from a common ancestor. Third, the PLTSs of some Proteobacteria are closely related with that of Bacteroidetes–Chlorobi. In the bacterial phylogeny, all Proteobacteria form a single group and Bacteroidetes–Chlorobi form another distinct and distantly related group. In the PLTS phylogeny, however, both Proteobacteria and Bacteroidetes–Chlorobi groups appear to have acquired their PLTS from a common ancestor. The ancestral PLTS of these groups is predicted to have originated from Bacteroidetes–Chlorobi, as this group remains in the same relative position in both the PLTS and bacterial trees. Further, it seems that the PLTS has been horizontally transferred from Chlorobi to Gammaproteobacteria because in the PLTS tree the *Saccharophagus degradans* (Gammaproteobacterium) is grouped with *Chlorobaculum parvum* (Chlorobi) and *Shewanella denitrificans* (Gammaproteobacterium) is grouped with *Bacteroides fragilis* (Bacteroidetes). Furthermore, incongruence between the two trees exists for *Deinococcus* although this could be due to low bootstrap value for this branch in the bacterial tree. Finally, a version of PLTS cluster with unique molecular characteristics, the Afp/PVC, which is found in Enterobacteriaceae, is also detected in a β-proteobacterium (*Burkholderia rhizoxinica*) but not in other β- or γ-proteobacteria (e.g., *Thauera*, *Saccharophagus*, and *Shewanella*). The isolated occurrence of this gene cluster therefore indicates a relatively recent genetic exchange.

## Conclusions

In this report, we describe the first wide-genome comparison study of gene clusters homologous to the recently described phage tail-like particle Afp/PVC, and we show that these clusters are not restricted to Gram-negative bacteria, but they are present in various bacterial phyla and Archaeal genomes. Phylogenetic analysis indicates that they have been acquired from the ancestors of the prokaryotic phyla and that they have been inherited directly to hundreds of microorganisms, although horizontal transfer is obvious in several cases. These clusters appear phylogenetically distinct from pyocins and T6SS; however, they do share some common molecular features with the latter, such as the presence of AAA+ ATPase and PAAR repeats or the lack of holins. These data suggest that these organisms may therefore also share important functionalities such as the capacity of toxin translocation to a target cell. They are thus referred to as PLTSs. The wide distribution of the PLTS among prokaryotes is a unique characteristic compared with the needle-like assemblies known to date and raises central questions related to the biology of this machinery, to its host/target range, and to the ecological implications of its introduction as a bacterial fitness determinant in mixed prokaryotic communities.

Previous studies on Afp/PVC revealed that the tail-like fiber machineries are more related to the adenovirus family than to bacteriophage-related tail fibers (Yang et al. 2006; Hurst et al. 2007), suggesting that PLTSs could have an affinity with eukaryotic cell surface components. These data also reveal the potential biotechnological applications of the PLTS as tool for “targeted protein translocation” in eukaryotic cells and an ecological biocontrol agent for targeted insect control. Further study of PLTS function and regulation in the context of various phylogenetically divergent prokaryotic species will provide insight for the functional specialization of this versatile machinery.

## Materials and Methods

The first result of the in-depth database genomic mining was the identification of entire phage-like secretion system (PLTS) gene cluster in the genomes of various and phylogenetically distant prokaryotes (fig. 1).

Complete annotated sequenced genomes were downloaded from the NCBI Genome database. Protein and nucleotide sequences from *S. entomophila* Afp cluster and *P. luminescens* PVCs were used for BLASTP, BLASTN, and reverse BLAST against various bacterial genomes in the NCBI, KEGG, and STRING databases. Only proteins showing the highest *E* value were retained. Clusters containing at least ten genes encoding proteins with similarity to known Afp–PVCs core proteins from *S. entomophila* and/or *P. luminescens* were considered as putative PLTS in the bacterial genomes examined. The genomic regions thus identified were examined within 4 kb up- and downstream for putative conserved genes associated with PLSS by reverse BLAST analysis against the *P. luminescens* genome. Maps of the genomic islands were constructed manually using the PowerPoint Microsoft office software (fig. 1). Proteins were identified by the corresponding locus numbers preceded by the organism’s name, strain number, or the abbreviation used in the text.

For the phylogenetic analysis, the amino acid sequences from five structural genes, which were common in the PLTS cluster among all prokaryotes examined were concatenated in a single sequence per organism. These genes were Sheath, G25, Baseplate, LysM, and VGRG. The concatenated amino acid sequences from 36 prokaryotes distributed across all major taxonomic groups were aligned using the Clustal-W program as implemented in the MEGA 5.0 package (Tamura et al. 2011), applying the standard parameters of the program. The phylogeny of the PLTS was reconstructed using the neighbor-joining method in MEGA 5.0. Distance was calculated using the poison model, assuming uniform substitution rates among sites. The trees were supported by 1,000 bootstraps.

Apart from the phylogeny of the PLTS, we reconstructed the core phylogeny of the 36 prokaryotes that contained the PLTS and used in this study. In 2009, Wu et al. described the phylogeny of 720 bacterial taxa based on the alignment of 31 concatenated gene products. From this list (kindly provided by Dr J. Eisen), we extracted the alignments for 33 of the bacteria used in our study. The three Archaea species, which were not present in the Wu et al. core phylogeny, were also excluded from our core-phylogenetic analysis based on the fact that in core phylogeny the Archaea is an outgroup to Bacteria. In case that an organism used for the PLTS phylogeny was not present in the Wu et al.’s list, we used sequences from a species of the same genus or family. This was the case for the Enterobacterium *Yersinia ruckeri* for which no sequence information existed in the Wu et al.’s publication, and, instead, we used its most closely relative, which was *Y. pseudotuberculosis* for the bacterial phylogeny reconstruction. In three cases, the closest relatives belonged to a different genus, that is, instead of *Microlunatus phosphovorus*, *Thauera* sp., and *Leeuwenhoekiella blandensis,* which we have used for the PLTS phylogeny, we used the aligned sequences of *Propionibacterium acnes, Azoarcus* sp., and *Capnocytophaga ochracea*, respectively, for the bacterial phylogeny reconstruction. In three cases (i.e., *Amycolatopsis mediterranei*, *Microcoleus* sp., and *Corallococcus coralloides*), the closest relatives were already included in the data set (i.e., *Actinosynnema mirum, Cyanothece* sp., and *Erythrobacter litoralis*), so they were excluded from the bacterial phylogeny. In total, we have extracted the aligned sequences of the 31 concatenated genes from 33 bacteria that they themselves, or their close relatives were included in the phylogeny of the PLTS and were present in the Wu et al.’s list. Based on these already aligned sequences, we reconstructed the phylogeny of the bacteria using the neighbor-joining method in MEGA 5.0. Distance was calculated using the poison model, assuming uniform substitution rates among sites. The trees were supported by 1,000 bootstraps.

## Note Added in Proof

Recently, it was demonstrated that phage tail-like structures produced by *Pseudoalteromonas luteoviolacea* and encoded by a gene cluster similar to PLTSs mediated *Hydroides elegans* metamorpfosis (Shikuma et al. 2014). Metamorphosis was induced after exposure to the bacterium producing the phage tail-like structures but not to the purified phage tail-like preparation. This is the first evidence of a beneficial for the eukaryotic partner interaction mediated by a PLTS structure.
